# Impact of public assistance on long-term outcomes after hospitalization for acute heart failure

**DOI:** 10.3389/fcvm.2026.1672844

**Published:** 2026-05-07

**Authors:** Takashi Morinaga, Masahiro Natsuaki, Akihiro Isotani, Kenji Kanenawa, Ko Yamamoto, Masato Fukunaga, Makoto Hyodo, Shinichi Shirai, Kenji Ando, Koichi Node

**Affiliations:** 1 Department of Cardiovascular Medicine, Saga University, Saga, Japan; 2 Department of Cardiology, Kokura Memorial Hospital, Kitakyushu, Japan

**Keywords:** Asia, heart failure, Japan, long-term outcome assessment, public assistance, socioeconomic status

## Abstract

**Background:**

Socioeconomic disparities adversely affect heart failure (HF) outcomes; however, the extent to which public assistance (PA) mitigates these differences remains uncertain.

**Methods and results:**

We retrospectively analyzed 596 patients who were discharged alive after hospitalization for acute HF between January and December 2015. The patients were classified according to their PA status. The primary outcome was a composite of all-cause death and HF rehospitalization. Secondary outcomes included individual components. Kaplan–Meier curves were used to compare unadjusted event rates, while Cox proportional hazards models adjusted for 25 clinical and sociodemographic covariates were used to evaluate associations in the full cohort. To confirm model robustness, 1:1 propensity score matching (PSM) was performed (*n* = 82 pairs) and sensitivity analyses were conducted in the matched cohort. The mean medical follow-up duration was 3.5 years. In the entire cohort, the primary outcome occurred in 69.6% of patients. During follow-up, the cumulative incidence of the primary outcome did not differ significantly between the PA and non-PA groups (log-rank test, *p* = 0.45). In the multivariable analysis, PA was not associated with a higher risk of the primary outcome (hazard ratio [HR], 1.06; 95% confidence interval [CI], 0.78–1.40; *p* = 0.68). Similar results were observed after PSM (HR, 0.87; 95% CI, 0.61–1.24; *p* = 0.46).

**Conclusion:**

PA was not associated with worse long-term outcomes after hospitalization for acute HF, suggesting that comprehensive public insurance coverage may help reduce socioeconomic disparities in HF care.

## Introduction

Heart failure (HF), a leading cause of hospitalization and mortality worldwide, affects individuals across all age groups (1, 2). Moreover, the burden of HF is expected to increase substantially as the worldwide population ages (3).

Socioeconomic disparities have been recognized as major determinants of premature mortality across various cardiovascular conditions (4). Several studies have reported worse outcomes in patients with HF and low socioeconomic status (SES), particularly in low- and lower–middle-income countries (5, 6). In these regions, limited access to guideline-directed medical therapy (GDMT) and follow-up care has been associated with significantly higher mortality rates and underuse of evidence-based treatments.

Importantly, SES disparities have been observed in other cardiovascular conditions. In Canada, a lower mean neighborhood income was associated with reduced access to revascularization and higher mortality rates after acute myocardial infarction (7). Similarly, in Taiwan, low-income patients exhibited significantly higher post-ST-segment elevation myocardial infarction mortality rates despite national health insurance coverage (8). These findings suggest that, even in universal healthcare systems, income-related disparities in cardiovascular outcomes may persist.

In Japan, the public assistance (PA) system is designed to ensure that individuals with limited financial resources receive medical services without out-of-pocket expense (9). This framework theoretically enables equitable access to HF care regardless of income. However, there is limited evidence on whether PA truly mitigates long-term disparities in HF outcomes. Most existing studies on PA and heart failure outcomes remain limited. One prior study reported no significant difference in 180-day outcomes between PA recipients and non-recipients but observed a higher risk of heart failure rehospitalization beyond 180 days among those receiving PA (10). To the best of our knowledge, no prior studies have examined the 5-year outcomes of patients with HF receiving PA using a matched cohort design.

This study investigated the long-term prognosis of patients with HF who were discharged alive after hospitalization for acute decompensated HF by comparing those receiving vs. not receiving PA. In Japan, the PA program serves as a proxy indicator of significant economic disadvantage and provides a framework for examining how institutional support may impact clinical outcomes. While our study does not directly compare groups based on SES, it aims to explore whether patients receiving PA exhibit different long-term outcomes after hospitalization for acute HF.

## Methods

### Study design and setting

This single-center retrospective observational analysis was conducted at Kokura Memorial Hospital, a tertiary acute-care facility in Kitakyushu, Japan. The study protocol was approved by the Institutional Review Board of Kokura Memorial Hospital (approval no. 19021201) and was conducted in accordance with the Declaration of Helsinki. Informed consent was obtained using an opt-out approach. Eligible patients were provided with written information regarding the study, and those who did not express a desire to opt out were included.

### Study population

**Figure 1:**
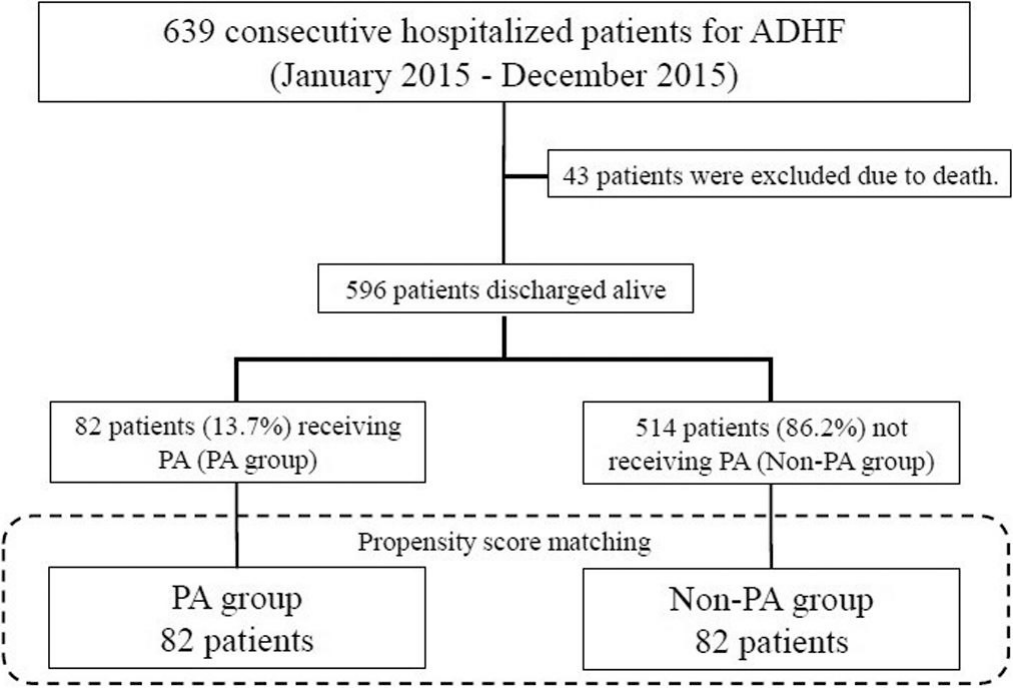
Study flow. ADHF, acute decompensated heart failure; PA, public assistance.

A total of 639 patients were hospitalized for acute HF between January and December 2015. Of them, 43 who died during hospitalization were excluded. The remaining 596 patients who were discharged were included in the primary analysis. PA status was used to classify patients into two groups. Subsequently, 1:1 propensity score matching (PSM) resulted in 82 matched pairs (*n* = 164) for the sensitivity analyses. The study design and patient selection process are shown in Figure 1.

Additionally, the PA group in this study refers to patients who were receiving Seikatsu-Hogo (livelihood protection) under the Japanese PA system at the time of admission. This system ensures minimum living standards and promotes self-sufficiency for those with financial difficulties. Once granted, PA is typically provided on a continuous basis, unless recipients voluntarily withdraw or become ineligible due to improvements in financial or health conditions. The duration is determined by the local welfare office based on periodic reassessments of individual needs. This program provides uniform coverage of medical expenses and living costs across all municipalities in Japan, based on the national standards set by the Ministry of Health, Labour and Welfare. Therefore, patients in the PA group received equivalent financial support in terms of healthcare access and daily living, and there were no differences in the scope of medical coverage among them. Eligibility for PA is determined by municipal welfare offices based on comprehensive assessments of household income, savings, family support availability, health status, and employability. Thus, patients who are socioeconomically disadvantaged but do not meet specific administrative criteria may not receive PA despite similar financial vulnerability.

### Exposure variable

The exposure of interest was the receipt of PA at the time of the index hospitalization. In Japan, the PA system covers all medical expenses for individuals with limited financial resources, eliminating their out-of-pocket payments and potentially improving their access to GDMT (11).

### Outcome measures

The primary outcome was a composite of all-cause death and HF rehospitalization after discharge. Secondary outcomes included all-cause death and HF rehospitalization, each of which was individually assessed. Time-to-event data for each outcome were evaluated using Kaplan–Meier survival curves and Cox proportional hazards regression models.

### Covariates and baseline variables

The patients' baseline characteristics, including demographics, comorbidities, and discharge medications, were collected from their electronic medical records. For the full cohort analysis, the multivariate Cox regression model included PA and 24 additional covariates marked with an asterisk (*) (Table 1). Variables included clinical characteristics such as age, sex, comorbid conditions (e.g., diabetes, atrial fibrillation, and chronic kidney disease), discharge medications [e.g., beta-blockers, angiotensin-converting enzyme inhibitors (ACEi) or angiotensin II receptor blockers (ARB), and mineralocorticoid receptor antagonists (MRA). These covariates were selected based on their known associations with HF prognosis and consistently used across the models.

**Table 1:** Baseline characteristics of study population.

Variables	Total	PA group	Non-PA group	*P* value
	(*N* = 596)	(*N* = 82)	(*N* = 514)	
Demographics and Anthropometrics				
Male sex*	266 (44.6%)	35 (42.6%)	231 (44.9%)	0.70
Age	76.6 ± 11.7	73.0 ± 11.6	77.2 ± 11.6	<0.001
Age ≥80 years*	285 (47.2%)	25 (30.4%)	260 (50.5%)	<0.001
Body weight, kg	52.7 ± 13.2	53.7 ± 15.1	52.5 ± 12.9	0.67
BMI	23.1 ± 4.3	23.7 ± 5.2	23.0 ± 4.1	0.35
BMI ≤ 22*	261 (43.7%)	35 (42.6%)	226 (43.9%)	0.24
Living Situation and Functional Status				
Lives alone*	161 (27.1%)	42 (51.2%)	120 (23.0%)	<0.001
Lives with family	399 (66.9%)	38 (46.3%)	361 (70.2%)	<0.001
Lives in care home	35 (5.8%)	2 (2.4%)	33 (6.4%)	0.11
Ambulatory	460 (77.1%)	65 (79.2%)	395 (76.8%)	0.62
Walks with cane	47 (7.8%)	8 (9.7%)	39 (7.5%)	0.51
Uses a wheelchair	59 (9.9%)	5 (6.1%)	54 (10.5%)	0.18
Bedridden*	30 (5.0%)	4 (4.8%)	26 (5.0%)	0.94
Etiology				
Ischemic heart disease*	238 (39.9%)	38 (46.3%)	200 (38.9%)	0.20
Dilated cardiomyopathy*	41 (6.8%)	5 (6.1%)	36 (7.0%)	0.75
Hypertensive heart disease	52 (8.7%)	11 (13.4%)	41 (7.9%)	0.12
Valvular heart disease	88 (14.7%)	8 (9.7%)	80 (15.5%)	0.14
Medical History				
Previous heart failure hospitalization*	219 (36.7%)	39 (47.5%)	180 (35.0%)	0.03
Previous myocardial infarction*	121 (20.3%)	23 (28.0%)	98 (19.0%)	0.06
Previous stroke	73 (12.2%)	9 (10.9%)	64 (12.4%)	0.70
Atrial fibrillation or flutter*	239 (40.1%)	28 (34.1%)	211 (41.0%)	0.23
VT or Vf	29 (4.8%)	5 (6.1%)	24 (4.6%)	0.58
COPD	44 (7.3%)	7 (8.5%)	37 (7.2%)	0.67
Asthma*	29 (4.8%)	5 (6.1%)	24 (4.6%)	0.58
Malignancy	71 (11.9%)	7 (8.5%)	64 (12.4%)	0.29
Dementia*	59 (9.9%)	5 (6.1%)	54 (10.5%)	0.18
Chronic kidney disease*	237 (39.7%)	34 (41.4%)	203 (39.4%)	0.73
Previous CABG	52 (8.7%)	3 (3.6%)	49 (9.5%)	0.05
Previous PCI*	179 (30.3%)	30 (36.5%)	149 (28.9%)	0.16
Pacemaker implantation	46 (7.7%)	5 (6.1%)	41 (7.9%)	0.54
ICD/CRT implantation*	40 (6.7%)	8 (9.7%)	32 (6.2%)	0.25
Hypertension	386 (64.7%)	50 (60.9%)	336 (65.3%)	0.44
Dyslipidemia	205 (34.4%)	30 (36.5%)	175 (34.0%)	0.65
Diabetes mellitus*	202 (33.8%)	38 (46.3%)	164 (31.9%)	0.01
Current smoker*	88 (14.7%)	19 (23.1%)	69 (13.4%)	0.028
Medication at discharge				
ACEi or ARB*	430 (72.1%)	63 (76.8%)	367 (71.4%)	0.30
Beta blocker*	450 (75.5%)	65 (79.2%)	385 (74.9%)	0.38
MRA*	299 (50.1%)	52 (63.4%)	247 (48.0%)	0.009
Loop diuretics*	488 (81.8%)	68 (82.9%)	420 (81.7%)	0.78
Tolvaptan	63 (10.5%)	11 (13.4%)	52 (10.1%)	0.38
Vitals at discharge				
Systolic blood pressure, mmHg	115 ± 18	113 ± 18	115 ± 18	0.20
SBP >140 mmHg	53 (8.9%)	8 (9.7%)	45 (8.7%)	0.82
SBP <90 mmHg	31 (5.2%)	6 (7.3%)	25 (4.8%)	0.58
Diastolic blood pressure	63 ± 12	63 ± 12	64 ± 12	0.94
Heart rate	68 ± 12	68 ± 12	69 ± 12	0.97
SpO_2_	97 ± 1	97 ± 1	97 ± 1	0.26
Symptoms at discharge				
Edema*	24 (4.0%)	5 (6.1%)	19 (3.7%)	0.33
Pleural effusion	71 (11.9%)	6 (7.3%)	65 (12.6%)	0.24
Pulmonary congestion	41 (6.8%)	7 (8.5%)	34 (6.6%)	0.56
Laboratory data at discharge				
LVEF, %	46 ± 15	44 ± 16	46 ± 15	0.30
rEF*	242 (40.6%)	37 (45.1%)	205 (39.8%)	0.38
Blood urea nitrogen, mg/dL	28 ± 15	29 ± 21	28 ± 14	0.82
Serum creatinine, mg/dL	1.5 ± 1.4	1.5 ± 1.4	1.5 ± 1.4	0.98
eGFR, mL/min/1.73 m^2^	43.6 ± 21.8	44.4 ± 21.5	43.5 ± 21.9	0.70
eGFR <30 mL/min/1.73 m^2^*	162 (27.1%)	21 (25.6%)	141 (27.4%)	0.72
Sodium, mEq/L	138 ± 3	139 ± 4	138 ± 3	0.49
Sodium < 130 mEq/L	11 (1.8%)	0 (0%)	11 (2.1%)	0.06
BNP, pg/mL	577 ± 993	709 ± 1,246	558 ± 955	0.80
Others				
Length of hospital stay, days	20 ± 18	19 ± 16	20 ± 19	0.52

Public assistance and the variables marked with an asterisk (*) were included as covariates in the multivariate Cox proportional hazards model.

ACEi, angiotensin-converting enzyme inhibitor; ARB, angiotensin II receptor blocker; BMI, body mass index; BNP, B-type natriuretic peptide; CABG, coronary artery bypass grafting; COPD, chronic obstructive pulmonary disease; CRT, cardiac resynchronization therapy; EF, left ventricular ejection fraction; eGFR, estimated glomerular filtration rate; ICD, implantable cardioverter-defibrillator; MRA, mineralocorticoid receptor antagonist; PA, public assistance; PCI, percutaneous coronary intervention; rEF, reduced ejection fraction (EF ≤ 40%); SBP, systolic blood pressure; SpO_2_, oxygen saturation; Vf, ventricular fibrillation; VT, ventricular tachycardia.

### Statistical analysis

Categorical variables are presented as frequencies and percentages and were compared using the chi-squared test. Continuous variables are expressed as mean and standard deviations and were compared using Student's *t*-test or the Wilcoxon rank-sum test according to their distributions.

For the entire cohort, Kaplan–Meier survival curves were generated to compare the time-to-event outcomes between the PA and non-PA groups, and differences were evaluated using the log-rank test. Multivariable Cox proportional hazards models were used to assess the association between PA and clinical outcomes with adjustment for the 25 covariates (12). To minimize confounding, PSM was performed as a sensitivity analysis using 1:1 nearest-neighbor matching without replacement based on a logistic regression model including the same covariates (13). Covariate balance pre- vs. post-PSM was assessed using standardized mean differences (SMDs) with an absolute value < 0.1 considered acceptable. A Love plot was used to visually assess the balance (14).

Of the matched cohort, Kaplan–Meier curves were constructed to compare the time-to-event outcomes between the PA and non-PA groups, and a Cox regression analysis was performed.

Subgroup analyses using the interaction terms were performed of the overall cohort to evaluate whether the association between PA and the primary outcome differed across clinically relevant strata.

Statistical significance was set at *p* < 0.05 for all analyses. All statistical analyses were performed using JMP version 12.2.0 (SAS Institute, Cary, NC, USA).

## Results

### Baseline characteristics of entire cohort

Table 1 summarizes the baseline characteristics of the entire cohort (*N* = 596). Patients receiving PA were generally younger, more likely to live alone, and had higher rates of previous hospitalization for HF and diabetes than those who did not receive PA. At discharge, the use of beta-blockers and ACEi or ARB was broadly similar between the two groups, whereas MRA use was significantly higher in the PA group (Table 1).

### Clinical outcomes of entire cohort

The mean medical follow-up duration was 3.5 years. During the follow-up period, the primary outcome (composite of all-cause death and HF rehospitalization) occurred in 415 of 596 patients (69.6%). All-cause death occurred in 306 patients (51.3%), while HF rehospitalization occurred in 274 patients (46.0%).

**Figure 2:**
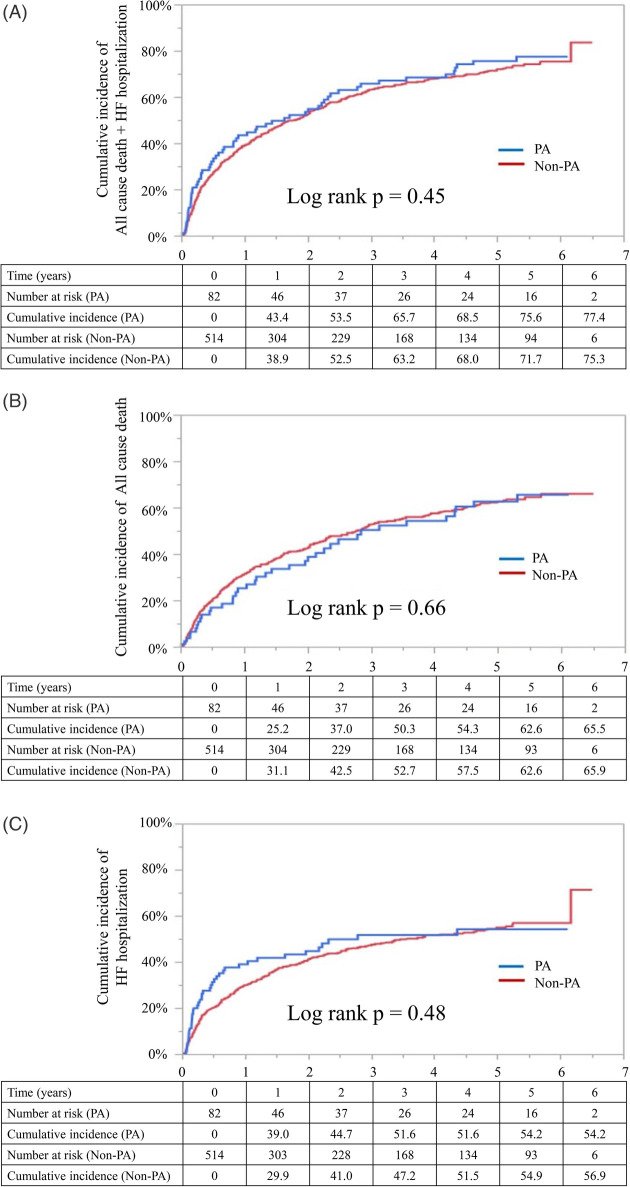
Kaplan–Meier curves for the entire cohort. Kaplan–Meier curves showing the cumulative incidence of the primary and secondary endpoints in the overall cohort (before propensity score matching). **(A)** The cumulative incidence of all-cause death and heart failure (HF) rehospitalization. **(B)** The cumulative incidence of all-cause death. **(C)** The cumulative incidence of HF rehospitalization. Blue lines represent those receiving public assistance (PA group), and red lines represent patients not receiving public assistance (Non-PA group). Log-rank *p*-values are shown for each comparison. The numbers at risk are listed below each panel. PA, public assistance; HF, heart failure.

Kaplan–Meier analyses demonstrated no significant differences in the cumulative incidence of any outcome between the PA and non-PA groups (Figure 2). Log-rank *p*-values were 0.45 for the composite outcome, 0.66 for all-cause death, and 0.48 for HF rehospitalization.

Multivariable Cox proportional hazards analyses adjusted for 25 covariates revealed that the PA status was not significantly associated with any outcome. The adjusted hazard ratio (HR) for the composite outcome was 1.06 [95% confidence interval (CI), 0.78–1.40; *p* = 0.68]; for all-cause death, the HR was 0.95 [(95% CI, 0.65–1.35; *p* = 0.80); and for HF rehospitalization, the HR was 1.05 (95% CI, 0.73–1.48; *p* = 0.76) (Table 2 and Supplementary Table S1).

**Table 2:** Crude and adjusted HRs of public assistance and clinical outcomes.

Outcome	Number of patients with events (cumulative incidence at 3.5 years)		Crude HR			Multivariable adjusted HR		
	PA (*n* = 82)	Non-PA (*n* = 514)	HR	95% CI	*P*	HR	95% CI	*P*
All-cause death + HF hospitalization	60 (67.1)	355 (65.6)	1.10	0.83–1.44	0.46	1.06	0.78–1.40	0.68
All-cause death	38 (52.3)	268 (55.3)	0.92	0.65–1.28	0.66	0.95	0.65–1.35	0.80
HF hospitalization	40 (51.6)	234 (49.8)	1.12	0.79–1.55	0.48	1.05	0.73–1.48	0.76

Hazard ratios (HRs) and 95% confidence intervals (CIs) of the association between public assistance (PA) and clinical outcomes in the full cohort (*N* = 596). Cox proportional hazards models were used to estimate crude and multivariate-adjusted HRs. The adjusted models included 25 clinical and sociodemographic covariates. The outcomes included a composite of all-cause death and hospitalization for heart failure (HF), all-cause death, and HF hospitalization.

PA, public assistance; HR, hazard ratio; CI, confidence interval; HF, heart failure.

### Baseline characteristics and clinical outcomes of matched cohort

After the PSM, the baseline characteristics were generally well-balanced between the PA and non-PA groups (Table 3). The SMDs for each covariate are illustrated in the Love plot (Supplementary Figure S1).

**Table 3:** Baseline characteristics after propensity score matching.

Variables	After PSM: Total (*N* = 164)	After PSM: PA group (*n* = 82)	After PSM: Non-PA group (*n* = 82)	*P* value	SMD after PSM
Demographics and Anthropometrics					
Male sex	68 (41.6%)	35 (42.6%)	33 (40.2%)	0.75	0.05
Age	73.2 ± 11.5	73.0 ± 11.6	73.5 ± 11.4	0.71	0.04
Age ≥80 years	53 (32.3%)	25 (30.4%)	28 (34.1%)	0.61	0.08
Body weight, kg	54.1 ± 14.5	53.7 ± 15.1	54.4 ± 13.9	0.67	0.05
BMI	23.3 ± 4.8	23.7 ± 5.2	23.0 ± 4.4	0.5	0.15
BMI ≤ 22	71 (43.2%)	35 (42.6%)	36 (43.9%)	0.87	0.03
Living Situation and Functional Status					
Lives alone	82 (50.0%)	42 (51.2%)	40 (48.7%)	0.75	0.05
Lives with family	80 (48.7%)	38 (46.3%)	42 (51.2%)	0.53	0.10
Lives in a care home	2 (1.2%)	2 (2.4%)	0 (0%)	0.15	0.22
Ambulatory	126 (76.8%)	65 (79.2%)	61 (74.3%)	0.45	0.12
Walks with a cane	17 (10.3%)	8 (9.7%)	9 (10.9%)	0.79	0.04
Uses a wheelchair	16 (9.7%)	5 (6.1%)	11 (13.4%)	0.11	0.25
Bedridden	5 (3.0%)	4 (4.8%)	1 (1.22%)	0.17	0.21
Etiology					
Ischemic heart disease	79 (48.1%)	38 (46.3%)	41 (50.0%)	0.63	0.07
Dilated cardiomyopathy	12 (7.3%)	5 (6.1%)	7 (8.5%)	0.54	0.09
Hypertensive heart disease	15 (9.1%)	11 (13.4%)	4 (4.8%)	0.05	0.30
Valvular heart disease	16 (9.7%)	8 (9.7%)	8 (9.7%)	1	0
Medical History					
Previous heart failure hospitalization	81 (49.3%)	39 (47.5%)	42 (51.2%)	0.63	0.07
Previous myocardial infarction	49 (29.8%)	23 (28.0%)	26 (31.7%)	0.6	0.08
Previous stroke	15 (9.1%)	9 (10.9%)	6 (7.3%)	0.41	0.13
Atrial fibrillation or flutter	57 (34.7%)	28 (34.1%)	29 (35.3%)	0.86	0.03
VT or Vf	12 (7.3%)	5 (6.1%)	7 (8.5%)	0.54	0.09
COPD	9 (5.4%)	7 (8.5%)	2 (2.4%)	0.08	0.27
Asthma	5 (3.0%)	5 (6.1%)	0 (0%)	0.02	0.36
Malignancy	15 (9.1%)	7 (8.5%)	8 (9.7%)	0.78	0.04
Dementia	10 (6.1%)	5 (6.1%)	5 (6.1%)	1	0
Chronic kidney disease	74 (45.1%)	34 (41.4%)	40 (48.7%)	0.34	0.15
Previous CABG	10 (6.1%)	3 (3.6%)	7 (8.5%)	0.18	0.21
Previous PCI	30 (36.5%)	30 (36.5%)	30 (36.5%)	1	0
Pacemaker implantation	10 (6.1%)	5 (6.1%)	5 (6.1%)	1	0
ICD/CRT implantation	17 (10.3%)	8 (9.7%)	9 (10.9%)	0.79	0.04
Hypertension	105 (64.0%)	50 (60.9%)	55 (67.0%)	0.41	0.13
Dyslipidemia	65 (39.6%)	30 (36.5%)	35 (42.6%)	0.42	0.12
Diabetes mellitus	66 (40.2%)	38 (46.3%)	28 (34.1%)	0.11	0.25
Current smoker	31 (18.9%)	19 (23.1%)	12 (14.6%)	0.16	0.22
Medication at discharge					
ACEi or ARB	124 (75.6%)	63 (76.8%)	61 (74.3%)	0.71	0.16
Beta blocker	130 (79.2%)	65 (79.2%)	65 (79.2%)	1	0
MRA	86 (52.4%)	52 (63.4%)	34 (41.4%)	<0.01	0.45
Loop diuretics	133 (81.1%)	68 (82.9%)	65 (79.2%)	0.54	0.09
Tolvaptan	19 (11.5%)	11 (13.4%)	8 (9.7%)	0.46	0.12
Vitals at discharge					
Systolic blood pressure, mmHg	114 ± 18	113 ± 18	115 ± 18	0.52	0.11
SBP >140 mmHg	15 (9.1%)	8 (9.7%)	7 (8.5%)	0.78	0.04
SBP < 90 mmHg	31 (5.2%)	6 (7.3%)	5 (6.1%)	0.75	0.05
Diastolic blood pressure	63 ± 12	63 ± 12	63 ± 13	0.77	0
Heart rate	69 ± 12	68 ± 12	69 ± 12	0.59	0.08
SpO_2_	97 ± 1	97 ± 1	97 ± 1	0.05	0
Symptoms at discharge					
Edema	6 (3.6%)	5 (6.1%)	1 (1.22%)	0.09	0.26
Pleural effusion	17 (10.3%)	6 (7.3%)	11 (13.4%)	0.27	0.20
Pulmonary congestion	14 (8.5%)	7 (8.5%)	7 (8.5%)	0.6	0
Laboratory data at discharge					
LVEF, %	43 ± 16	44 ± 16	43 ± 16	0.68	0.06
rEF	77 (46.9%)	37 (45.1%)	40 (48.7%)	0.63	0.07
Blood urea nitrogen, mg/dL	28 ± 17	29 ± 21	28 ± 13	0.61	0.06
Serum creatinine, mg/dL	1.7 ± 1.5	1.5 ± 1.4	1.9 ± 1.7	0.19	0.26
eGFR, mL/min/1.73 m^2^	42.6 ± 22.9	44.4 ± 21.5	40.8 ± 24.2	0.22	0.16
eGFR < 30 mL/min/1.73 m^2^	50 (30.4%)	21 (25.6%)	29 (35.7%)	0.17	0.22
Sodium, mEq/L	138 ± 3	139 ± 4	138 ± 3	0.84	0.28
Sodium < 130 mEq/L	1 (0.6%)	0 (0%)	1 (1.2%)	0.31	0.16
BNP, pg/mL	719 ± 1,219	709 ± 1,246	726 ± 1,222	0.39	0.01
Others					
Length of hospital stay, days	21 ± 20	19 ± 16	22 ± 24	0.9	0.15

ACEi, angiotensin-converting enzyme inhibitor; ARB, angiotensin II receptor blocker; BMI, body mass index; BNP, B-type natriuretic peptide; CRT, cardiac resynchronization therapy; COPD, chronic obstructive pulmonary disease; CABG, coronary artery bypass grafting; eGFR, estimated glomerular filtration rate; ICD, implantable cardioverter-defibrillator; LVEF, left ventricular ejection fraction; MRA, mineralocorticoid receptor antagonist; PA, public assistance; PCI, percutaneous coronary intervention; PSM, propensity score matching; SBP, systolic blood pressure; SMD, standardized mean difference; SpO_2_, oxygen saturation; rEF, reduced ejection fraction (≤40%); Vf, ventricular fibrillation; VT, ventricular tachycardia.

After the PSM, 164 patients (82 pairs) were included in the study. The primary outcome was observed in 60 patients (73.1%) in the PA group vs. 64 patients (78.0%) in the non-PA group. All-cause death occurred in 38 (46.3%) and 43 (52.4%) patients, while HF rehospitalization occurred in 40 (48.7%) and 46 (56.0%) patients.

**Figure 3:**
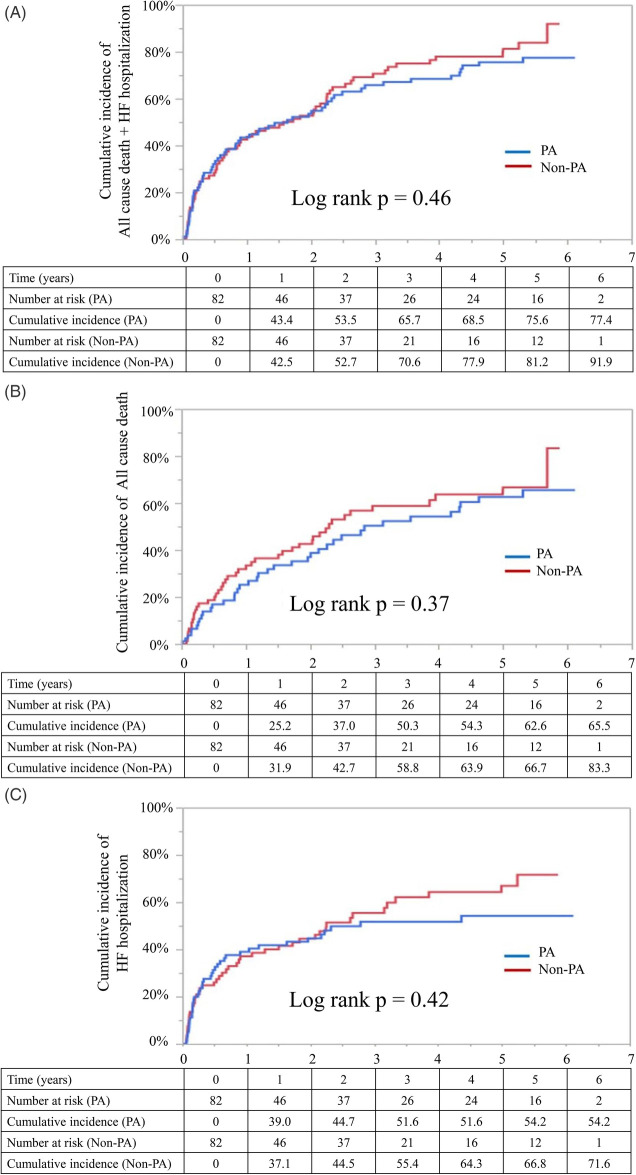
Kaplan–Meier curves for the propensity score–matched cohort. Kaplan–Meier curves showing the cumulative incidence of the primary and secondary endpoints in the propensity score–matched cohort. **(A)** The cumulative incidence of all-cause death and heart failure (HF) rehospitalization. **(B)** The incidence of all-cause death. **(C)** The incidence of HF rehospitalization. Blue lines represent those receiving public assistance (PA group), and red lines represent patients not receiving public assistance (Non-PA group). Log-rank *p*-values are shown for each comparison. The numbers at risk are listed below each panel. PA, public assistance; HF, heart failure.

The Kaplan–Meier analysis showed no significant intergroup differences in the cumulative incidence of any outcome. Log-rank *p*-values were 0.46 for the primary outcome, 0.37 for all-cause death, and 0.42 for HF rehospitalization (Figure 3).

A Cox proportional hazards regression of the matched cohort demonstrated no significant association between PA status and outcomes (composite outcome: HR, 0.87; 95% CI, 0.61–1.24; *p* = 0.46; all-cause death: HR, 0.78; 95% CI, 0.49–1.22; *p* = 0.28; and HF rehospitalization: HR, 0.84; 95% CI, 0.54–1.28; *p* = 0.42) (Supplementary Table S2).

### Subgroup analyses

Subgroup analyses of the entire cohort demonstrated no significant interaction between PA status and outcomes across multiple predefined clinical strata (Supplementary Figure S2). The absence of an effect modification supports the consistency of the findings across different patient groups.

## Discussion

In this study of patients discharged alive after hospitalization for acute HF, we found that the receipt of PA was not associated with worse long-term outcomes, including all-cause mortality and HF rehospitalization. These findings hold true in the full cohort, after the adjustment for 25 clinical and sociodemographic covariates, and in a PSM cohort in which patients receiving and not receiving PA had comparable event rates. Subgroup analyses revealed no significant interactions between PA status and clinical outcomes across multiple patient strata. Collectively, our findings suggest that the Japanese PA system may effectively mitigate the adverse effects of socioeconomic vulnerability on long-term HF prognosis.

SES is a widely recognized determinant of cardiovascular health. Multiple studies reported that patients with a low SES experience poorer HF outcomes owing to delayed access to care, underuse of GDMT, and limited follow-up continuity (4–6). Importantly, a recent global registry showed that patients in low- and lower-middle-income countries had markedly lower GDMT usage and higher mortality rates than those in high-income countries (6). These disparities are thought to be driven by systemic factors, including healthcare infrastructure limitations and economic barriers to treatment (15).

Our study adds to the growing body of literature suggesting that when financial barriers are removed and healthcare access is equitable, long-term outcomes in vulnerable populations may improve (16). The Japanese PA system fully subsidizes medical costs, including those for evidence-based HF medications. This universal access likely contributed to the broadly similar availability of key therapies—such as beta-blockers and ACEi or ARB—observed in both groups, although MRA use was significantly higher in the PA group. One possible explanation is that PA recipients, whose medical expenses are fully covered, may have fewer financial concerns about potential adverse effects such as hyperkalemia or renal dysfunction, which could otherwise discourage MRA prescription. Additionally, physicians may be more willing to initiate MRAs in PA patients given their better access to follow-up care and laboratory monitoring under the PA system. As demonstrated in previous trials and real-world registries, the use of such therapies is strongly associated with improved HF prognosis (17, 18).

In interpreting these findings, it is important to consider the broader healthcare context in Japan. While public assistance (PA) status may serve as a proxy for low SES, SES is not synonymous with healthcare equity. Japan's universal health insurance system ensures that nearly all residents, regardless of income, have access to healthcare at relatively low cost. Furthermore, a standardized fee schedule, high physician density, and broad geographic distribution of healthcare facilities help to promote equity in care delivery. These structural features may have mitigated SES-related disparities in this cohort.

Similar observations have been reported in other chronic diseases in Japan. For example, although lower SES is associated with worse outcomes in conditions such as diabetes or cancer, the magnitude of disparities tends to be smaller than in countries with higher out-of-pocket costs (19, 20). These contextual factors suggest that the favorable outcomes observed in PA recipients may be partially attributed to Japan's health system design, in addition to the direct effects of PA coverage.

Conversely, GDMT underuse or discontinuation has been associated with substantial increases in mortality and morbidity rates (21, 22). In this context, our findings support the notion that public insurance systems with robust drug coverage may act as socioeconomic equalizers in chronic disease management (16, 23). Notably, although the PA recipients in our study had a higher burden of clinical vulnerability, such as prior HF hospitalization, diabetes, and solitary living, their long-term outcomes did not differ significantly from those of their more socially advantaged peers.

While our findings suggest a long-term benefit of PA on clinical outcomes, a prior study reported an increased risk of HF rehospitalization beyond 180 days among PA recipients (10). This discrepancy may be attributed to differences in study design, sample size, follow-up duration, outcome definitions, and model specifications. For example, while both studies adjusted for clinical and social factors, differences in how these were measured or operationalized (e.g., discharge medications, living arrangements) may have contributed to divergent results.

Importantly, our study extended the current literature by examining a long-term follow-up period. While many previous investigations focused on short-term outcomes, often limited to hospitalization or 1-year mortality, HF is a lifelong condition that requires sustained interactions with the healthcare system (10). Socioeconomic disparities in outcomes may not be fully apparent in the early phases but can emerge or widen over time. Our study, which included follow-up data for up to 6.5 years (median, 3.5 years), provides a more comprehensive assessment of long-term prognosis. The observed equivalence in outcomes between the PA and non-PA groups suggests that Japan's PA system may effectively offset the baseline disadvantages faced by low-income patients.

## Limitations

This study had several limitations. First, this was a retrospective, single-center study, which may have limited the generalizability of its results. However, the inclusion of a large number of covariates and use of robust statistical methods (e.g., multivariable Cox regression and PSM) help mitigate confounding factors. Second, the PA status was determined only at discharge, and we lacked information on patients' PA eligibility prior to admission or any changes post-discharge. This introduces a potential for exposure misclassification, as PA status may have changed over time. However, in the context of Japan's long-term PA provision, such misclassification is likely to be non-differential and may bias results toward the null. Additionally, the potential for selection bias cannot be excluded, as patients who receive PA may differ from those who are eligible but not enrolled due to administrative or social factors. Future studies incorporating direct measures of SES could further clarify this issue. Third, although we used PA status as a proxy for a low SES, SES is a multidimensional construct that includes income, education, occupation, and social support, which was not fully captured. Our study could not include a control group composed exclusively of patients with socioeconomic disadvantage but without PA, nor did we match groups based on SES itself. Therefore, while our findings suggest that PA is associated with better long-term outcomes, we cannot conclusively determine whether PA directly mitigates disparities in care among socioeconomically vulnerable populations. Moreover, our study did not capture non-financial barriers that may affect healthcare access, such as health literacy, care coordination, or availability of specialty services. These unmeasured factors may have contributed to the observed outcomes. Further research is warranted to disentangle these effects in cohorts explicitly stratified by SES and with more comprehensive measures of SES. Fourth, residual confounding factors cannot be entirely excluded, particularly unmeasured variables such as medication adherence or frailty. Fifth, although GDMT use at discharge was documented, data on medication titration and longitudinal adherence were unavailable. Finally, our study cohort was based in Japan, where the PA system provides comprehensive health coverage. Therefore, its results may not be generalizable to healthcare systems with different social support structures.

## Conclusion

In this study, PA was not associated with worse long-term outcomes after hospitalization for acute HF, suggesting that comprehensive public insurance coverage may help reduce socioeconomic disparities in HF care.

## Data Availability

The raw data supporting the conclusions of this article will be made available by the authors, without undue reservation.
