# Supplementary material for: Impact of public assistance on long-term outcomes after hospitalization for acute heart failure
Source: Front Cardiovasc Med. 2026 May 7;13:1672844. doi: 10.3389/fcvm.2026.1672844 (PMC13189743; doi:10.3389/fcvm.2026.1672844)
Supplement: Supplementary file 1 [file Datasheet1.docx]

**Supplementary Tables and Figures**

**Supplementary Table 1. Multivariable Cox Proportional Hazards Model of Primary Outcome of Entire Cohort**

|  | Univariate analysis | | | Multivariate analysis | | |
| --- | --- | --- | --- | --- | --- | --- |
|  | HR | 95%CI | P | HR | 95%CI | P |
| Public assistance | 1.10 | 0.83-1.44 | 0.46 | 1.06 | 0.78-1.40 | 0.68 |
| Male sex | 1.20 | 0.99-1.46 | 0.06 | 1.21 | 0.98-1.50 | 0.07 |
| Age ≥ 80 years | 1.49 | 1.23-1.81 | < 0.01 | 1.40 | 1.13-1.74 | < 0.01 |
| BMI ≤ 22 | 0.94 | 0.77-1.14 | 0.58 | 0.95 | 0.77-1.18 | 0.70 |
| Current smoker | 0.87 | 0.65-1.15 | 0.35 | 1.13 | 0.83-1.51 | 0.40 |
| Lives alone | 0.97 | 0.78-1.21 | 0.85 | 1.02 | 0.81-1.28 | 0.81 |
| Bedridden | 1.14 | 0.71-1.73 | 0.54 | 1.17 | 0.71-1.82 | 0.51 |
| Ischemic heart disease | 1.14 | 0.94-1.39 | 0.16 | 0.77 | 0.59-1.01 | 0.06 |
| Dilated cardiomyopathy | 0.81 | 0.52-1.19 | 0.30 | 1.01 | 0.62-1.56 | 0.96 |
| Previous heart failure hospitalization | 1.71 | 1.40-2.08 | < 0.01 | 1.31 | 1.05-1.63 | 0.01 |
| Previous myocardial infarction | 1.70 | 1.35-2.13 | < 0.01 | 1.31 | 0.96-1.78 | 0.08 |
| Atrial fibrillation or flutter | 1.23 | 1.01-1.50 | 0.03 | 1.25 | 1.01-1.55 | 0.03 |
| Asthma | 1.08 | 0.67-1.65 | 0.71 | 0.89 | 0.54-1.38 | 0.62 |
| Dementia | 1.46 | 1.06-1.97 | 0.02 | 1.35 | 0.96-1.86 | 0.08 |
| Chronic kidney disease | 1.95 | 1.61-2.38 | < 0.01 | 1.45 | 1.13-1.84 | < 0.01 |
| Previous PCI | 1.72 | 1.40-2.10 | < 0.01 | 1.50 | 1.13-1.98 | < 0.01 |
| ICD/CRT implantation | 1.98 | 1.38-2.75 | < 0.01 | 1.56 | 1.04-2.27 | 0.03 |
| Diabetes mellitus | 1.12 | 0.91-1.37 | 0.25 | 0.98 | 0.78-1.23 | 0.90 |
| ACEi or ARB at discharge | 0.87 | 0.70-1.07 | 0.20 | 0.79 | 0.63-0.99 | 0.04 |
| Beta blocker at discharge | 0.94 | 0.76-1.17 | 0.61 | 0.88 | 0.69-1.12 | 0.30 |
| MRA at discharge | 0.78 | 0.65-0.95 | 0.01 | 0.98 | 0.78-1.23 | 0.89 |
| Loop diuretics at discharge | 1.06 | 0.83-1.38 | 0.60 | 1.01 | 0.77-1.35 | 0.90 |
| Lower-limb edema at discharge | 1.96 | 1.21-2.99 | < 0.01 | 1.35 | 0.80-2.16 | 0.23 |
| rEF | 0.88 | 0.72-1.07 | 0.20 | 1.00 | 0.79-1.26 | 0.98 |
| eGFR < 30 mL/min/1.73 m^2^ at discharge | 1.87 | 1.51-2.29 | < 0.01 | 1.35 | 1.04-1.75 | 0.02 |

Multivariate Cox proportional hazards analysis of the association between predefined clinical variables and composite outcomes (all-cause death and heart failure rehospitalization) in the overall cohort.

Abbreviations: ACEi, angiotensin-converting enzyme inhibitor; ARB, angiotensin II receptor blocker; BMI, body mass index; CI, confidence interval; CRT, cardiac resynchronization therapy; eGFR, estimated glomerular filtration rate; HR, hazard ratio; ICD, implantable cardioverter-defibrillator; MRA, mineralocorticoid receptor antagonist; PCI, percutaneous coronary intervention; rEF, reduced ejection fraction (≤40%)

**Supplementary Table 2. Cox Proportional Hazards Analysis of Matched Cohort**

|  | Number of patient with events (cumulative incidence at 3.5 years) | | HR | 95% CI | P value |
| --- | --- | --- | --- | --- | --- |
| Outcome | PA (n = 82) | non-PA (n = 82) |  |  |  |
| All-cause death + HF hospitalization | 60 (67.1) | 64 (75.0) | 0.87 | 0.61-1.24 | 0.46 |
| All-cause death | 38 (52.3) | 43 (58.8) | 0.78 | 0.49-1.22 | 0.28 |
| HF hospitalization | 40 (51.6) | 46 (62.1) | 0.84 | 0.54-1.28 | 0.42 |

Cox proportional hazards analysis evaluating association between PA and composite outcomes in propensity score–matched cohort

Abbreviations: CI, confidence interval; HF, heart failure; HR, hazard ratio; PA, public assistance

**Supplementary Figure 1. Love plot showing covariate balance before and after PSM**


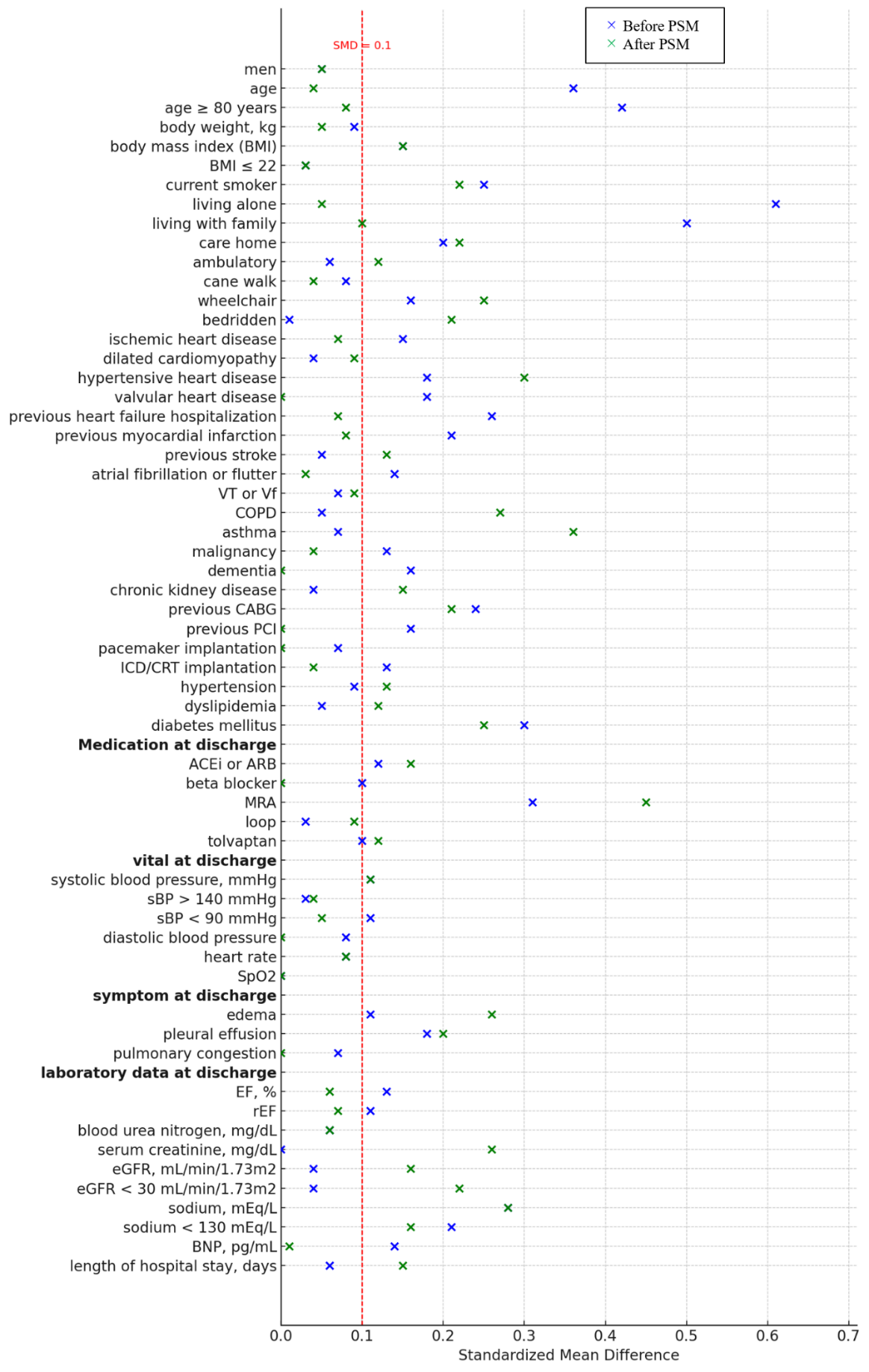


Standardized mean differences before versus after PSM for baseline variables. A threshold of ±0.1 was used to indicate acceptable covariate balance. Overlapping markers indicate identical SMD before and after PSM.

Abbreviations: ACEi, angiotensin-converting enzyme inhibitor; ARB, angiotensin II receptor blocker; BMI, body mass index; BNP, B-type natriuretic peptide; CABG, coronary artery bypass grafting; COPD, chronic obstructive pulmonary disease; CRT, cardiac resynchronization therapy; LVEF, left ventricular ejection fraction; eGFR, estimated glomerular filtration rate; ICD, implantable cardioverter-defibrillator; MRA, mineralocorticoid receptor antagonist; PCI, percutaneous coronary intervention; PSM, propensity score matching; rEF, reduced ejection fraction (≤40%); SBP, systolic blood pressure;; SMD, standardized mean difference; SpO_2_, oxygen saturation; Vf, ventricular fibrillation; VT, ventricular tachycardia

**Supplementary Figure 2. Forest plot of subgroup analysis evaluating association between public assistance and primary composite outcomes**


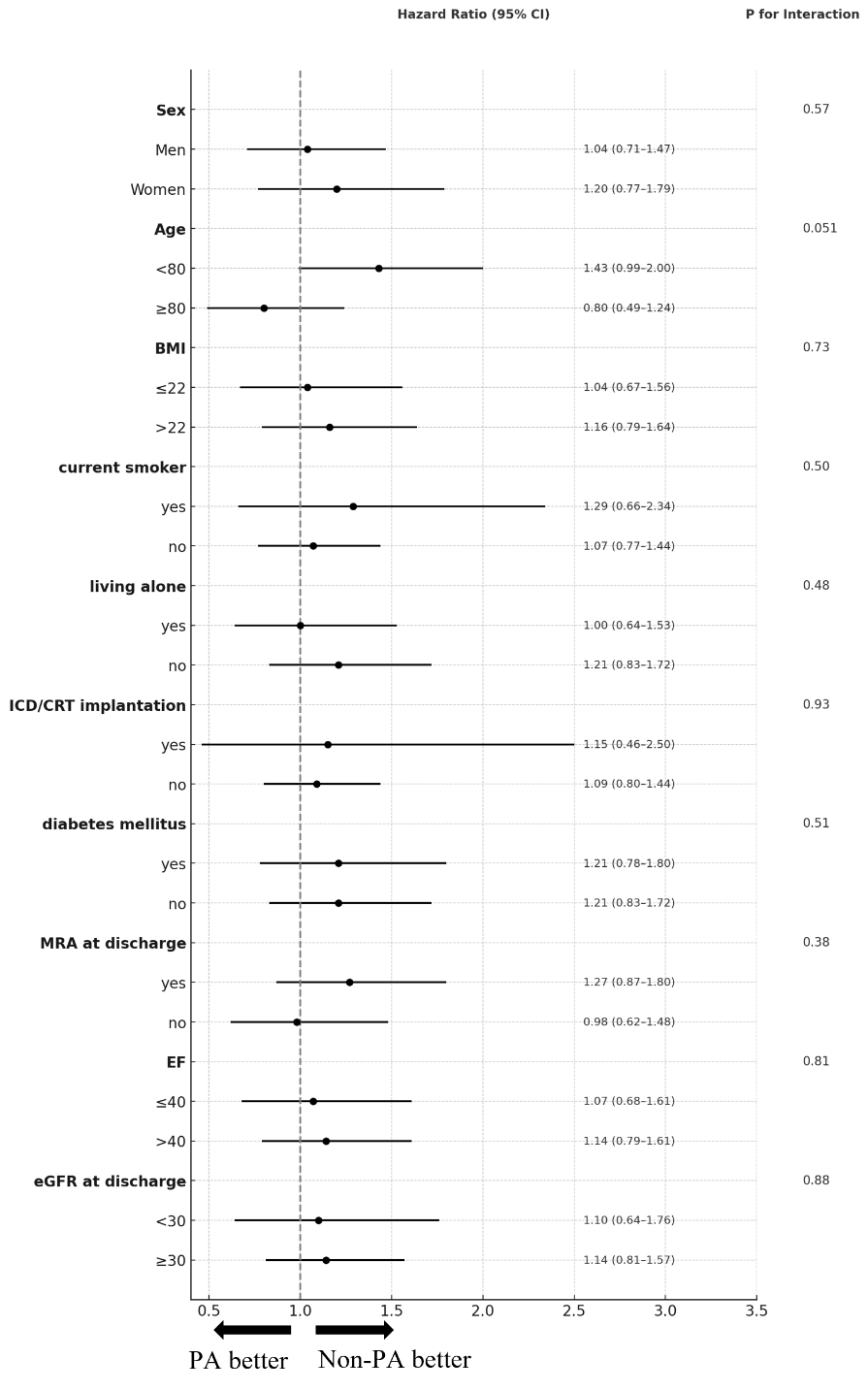


Forest plot illustrating the hazard ratios (HRs) and 95% confidence intervals (CIs) of the association between public assistance (PA) and composite outcomes (all-cause mortality and heart failure rehospitalization) across predefined subgroups. Interaction p-values are shown to assess the effect modification.

Abbreviations: BMI, body mass index; CRT, cardiac resynchronization therapy; EF, ejection fraction; eGFR, estimated glomerular filtration rate; ICD, implantable cardioverter-defibrillator; MRA, mineralocorticoid receptor antagonist
